# Understanding the Relationship Between Alcohol Consumption and HIV Risk Behaviors in U.S. Adolescents: A Systematic Review of Youth Risk Behavior Survey Findings (2005–2021)

**DOI:** 10.3390/healthcare13182370

**Published:** 2025-09-21

**Authors:** Shahin Davoudpour, Madeline Kerr, Gregory L. Phillips II

**Affiliations:** 1 Department of Medical Social Sciences, Northwestern University Feinberg School of Medicine, Chicago, IL 60611, USA; 2 Department of Sociology, University of California—Berkeley, Berkeley, CA 94720, USA

**Keywords:** adolescent health, alcohol use, HIV risk behaviors, YRBS data, systematic review

## Abstract

**Background/Objectives**: Alcohol use is a significant public health concern for adolescents, not only for its direct health impacts but also for its association with other health risk behaviors. In particular, alcohol use has been linked to sexual behaviors that may increase the risk of HIV transmission. This systematic review aims to provide a deeper understanding of the relationships between various alcohol- and HIV-related risk behaviors among adolescents by synthesizing existing literature that exclusively uses Youth Risk Behavior Survey (YRBS) data. **Methods**: This review followed the Preferred Reporting Items for Systematic Reviews and Meta-Analyses (PRISMA) guidelines. A search was conducted in PubMed, Web of Science, and PsycINFO. Eligible studies were peer-reviewed, published in English, and analyzed associations between alcohol use (age at first drink, alcohol use, binge drinking) and HIV risk behaviors (number of sexual partners, condom use, HIV testing) using YRBS data from the 2005–2021 collection years. Two authors independently screened 1133 records for eligibility, with 17 studies included in the final review. **Results**: The included studies consistently found a significant positive association between the frequency of alcohol use and binge drinking and a greater number of sexual partners. However, the evidence for an association between alcohol use and condom non-use was mixed. Limited evidence suggests that age of first alcohol use is not correlated with condom non-use. A key finding was the wide variation in study methodology, including the use of lifetime versus recent and dichotomized versus ordinal measures. Additionally, few studies were guided by a theoretical framework. **Conclusions**: The findings support a consistent link between alcohol use and having a greater number of sexual partners but highlight mixed evidence regarding condom use and little evidence for an association with HIV testing. This review demonstrates a need for more nuanced, theory-driven analyses that better utilize the multidimensional data available in the YRBS to capture the complex nature of risk behaviors.

## 1. Introduction

Adolescence represents a critical developmental epoch, characterized by profound biological, psychological, and social transformations that shape an individual’s trajectory into adulthood. Concurrently, this period is marked by the onset of many risk behaviors that can precipitate significant adverse health outcomes [1]. Alcohol use stands as a particularly salient public health concern, given its prevalence among youth; an estimated 22.1% of U.S. adolescents reported current use of alcohol in 2023 [2]. Alcohol also has well-established associations with an array of detrimental consequences, including an increase in the likelihood of engaging in health risk behaviors related to HIV and other sexually transmitted infections [3,4,5]. This concern is further complicated by the ambiguous nature of alcohol in society; while legal and socially integrated for adults, its consumption is strictly prohibited for minors, creating a nuanced context for understanding and addressing youth engagement in alcohol use. Consequently, a comprehensive understanding of the intricate nexus between adolescent alcohol consumption and HIV risk is imperative for shaping public health interventions and informing clinical practices that support adolescents’ behavioral health.

While recent epidemiological trends show gradual decreases in the early initiation of alcohol consumption among youth [6,7], youth exposure to alcohol is still common and multifaceted in its consequences. Beyond its direct physiological impacts, such as potential hepatotoxicity [8] and impaired neurodevelopment [9], early and substantial alcohol use can influence risk-taking behaviors. The inherent disinhibiting properties of alcohol can compromise executive functions, leading to impaired judgment and decision-making and potentially increasing the propensity for behaviors that elevate the risk of HIV transmission [10,11]. These behaviors encompass, but are not limited to, condomless sex, having multiple sexual partners, engaging in exchange sex, and using drugs before or during sex. Given the potential for severe, long-term health ramifications, a granular understanding of how alcohol use specifically intersects with these HIV-related risk behaviors is foundational for developing evidence-based public health policies and precisely targeted interventions designed to promote the health and well-being of young populations.

The Youth Risk Behavior Surveillance System (YRBSS), a robust and nationally representative data collection endeavor administered by the U.S. Centers for Disease Control and Prevention (CDC), has been indispensable in monitoring a broad spectrum of health risk behaviors among adolescents across the United States. The YRBS provides invaluable longitudinal and cross-sectional insights into trends concerning alcohol use and its associated risk behaviors across diverse demographic strata [2]. Despite the system’s extensive utility and the voluminous data it yields on adolescent health, there is a paucity of systematic reviews exclusively leveraging YRBS data to explore the relationship between adolescent alcohol consumption and HIV-related risk-taking behaviors. While individual investigations have utilized YRBS to examine specific facets of this relationship, a consolidated, systematic synthesis of these studies remains elusive. This absence precludes a comprehensive appreciation of prevailing methodological and theoretical approaches and patterns of key findings within this specific domain of research. To ameliorate this critical gap, this systematic review aims to provide a comprehensive synthesis of investigations that exclusively utilize YRBS data collected between 2005 and 2021 to examine the relationship between alcohol consumption and HIV-related risk behaviors among adolescents.

More specifically, this review aims to (1) offer a consolidated overview of the extant evidence base regarding the association between alcohol use and HIV risk behaviors in youth, as derived exclusively from YRBS data; (2) identify consistent associations and areas of divergence across studies, thereby fostering a more nuanced understanding of these relationships; (3) critically evaluate the methodological approaches and theoretical frameworks employed in previous YRBS-based research, providing insights for guiding future investigations in this vital field. Ultimately, through a rigorous and systematic examination of the existing YRBS literature, this review seeks to deepen our understanding of a paramount public health challenge and contribute meaningfully to the development of more effective, evidence-based prevention strategies and interventions designed to promote healthier outcomes for young people.

## 2. Method

We followed the most recent Preferred Reporting Items for Systematic Reviews and Meta-Analyses (PRISMA) guidelines throughout the review process [12]. We developed a protocol for this review in accordance with the PRISMA-P guideline [13] and registered the protocol online in the PROSPERO database (registration ID: CRD42024550748).

## 3. Information Sources and Search Strategy

We retrieved literature from PubMed, Web of Science, and PsycINFO databases. S.D. and M.K. collaboratively developed a Boolean search strategy including terms related to alcohol, risk-taking behavior, and YRBS. The search strategy for all three databases reads as follows: (“risk behavior*” OR “risky behavior*” OR “risk taking” OR “risk-taking”) AND (alcohol* OR drink*) AND (“YRBS” OR “youth risk behavior surveillance” OR “youth risk behavior survey”). Filters were used to limit search results to peer-reviewed journal articles, and no other filters or restrictions (e.g., by publication date) were applied at this stage. M.K. conducted searches in all databases on 10 April 2024. The study selection and data analysis processes took place from April to June 2024.

### 3.1. Eligibility Criteria

For inclusion in the review, studies were required to be peer-reviewed journal articles, published in English, and report secondary analyses of data from the CDC’s YRBS. We excluded reviews, conceptual articles, and other works that did not present original analyses of YRBS data. We also excluded studies that used data from versions of the YRBS administered outside the United States or that adapted YRBS measures to collect original data separately from the CDC’s official surveillance system.

Studies had to include one or more specified alcohol use behaviors and one or more HIV risk behaviors as variables. Alcohol use variables included age at first drink (measured through the YRBS question “How old were you when you had your first drink of alcohol other than a few sips?”), any/recent alcohol use (“During the past 30 days/during your life, on how many days did you have at least one drink of alcohol?”), and binge drinking (“During the past 30 days, on how many days did you have 5 or more drinks of alcohol in a row, that is, within a couple of hours?”). HIV risk behaviors included number of sexual partners (“During the past 3 months/during your life, with how many people did you have sexual intercourse?”), condom use (“The last time you had sexual intercourse, did you or your partner use a condom?”), and HIV testing (“Have you ever been tested for HIV, the virus that causes AIDS?”). Additionally, studies were required to analyze associations between alcohol use and HIV risk behaviors; therefore, we excluded studies that only presented descriptive statistics or only examined these outcomes separately in relation to other variables.

Finally, we restricted our review to studies that used YRBS data from the 2005–2021 collection years, including studies published after 2021 that used data collected within these years. The decision to include studies utilizing YRBS data from 2005 onward was driven by significant methodological advancements in the survey. Beginning in 2005, the YRBS initiated the collection of data on sexual identity, allowing for the disaggregation and analysis of health-related behaviors among sexual minority adolescents [14]. While our primary aim was not focused on this subpopulation, the availability of these data from 2005 onwards has profound implications for understanding the intersection of alcohol consumption and HIV-related risk behaviors across diverse adolescent groups. Prior to 2005, nuanced analyses by sexual identity were not possible with YRBS data, limiting opportunities for comprehensive investigations into disparities and specific risk factors relevant to this population. Therefore, our chosen timeframe ensures that the synthesized evidence reflects a period where studies could, and increasingly did, account for this demographic variable, contributing to a more complete and contemporary understanding of adolescent risk behaviors.

There were few changes to YRBS questions about our variables of interest that would complicate comparisons of findings across the review timeframe [15]. The wording of survey questions and response options remained the same for age at first drink, recent alcohol use, binge drinking, recent and lifetime sexual partners, and HIV testing. However, beginning in 2019, YRBS began to exclusively measure current alcohol use (i.e., in the past 30 days) and not lifetime alcohol use [16]. Thus, it is only possible to examine associations between lifetime alcohol use and HIV risk behaviors using YRBS data collected before 2019.

### 3.2. Selection Process

The search results from all databases were uploaded to EndNote reference management software and de-duplicated using EndNote’s duplicate identification feature. The unique records, including titles, key words, and abstracts, were transferred to a Microsoft Excel spreadsheet. For the first phase of screening, M.K. independently reviewed the titles and abstracts of each record against the eligibility criteria and documented the inclusion or reasons for exclusion for each record on the Excel spreadsheet. Full texts were retrieved for all records that met eligibility criteria at this stage. S.D. and M.K. then used separate spreadsheets to independently screen each full text against the same criteria. After completing this phase, S.D. and M.K. met to compare their decisions regarding inclusion or reasons for exclusion for each text. S.D and M.K. resolved disagreements through discussion amongst themselves and recorded the final decisions on a shared document.

### 3.3. Data Extraction

Studies included in the final review were uploaded to a new spreadsheet including a standardized data extraction form. M.K. performed the data extraction for all studies, and S.D. checked the extracted information for accuracy. There were no disagreements at this stage. For each study, the extracted data included the research aims, theoretical frameworks, years and levels (e.g., national, state) of YRBS data examined, whether the data were pooled across years and/or sites of collection, and the examined alcohol use and HIV risk variables. Studies’ target populations, any additional inclusion/exclusion criteria imposed on the samples, and sample sizes and age ranges were also extracted. Finally, studies’ statistical analysis methods, operationalizations of “risk behavior,” and findings regarding relationships between alcohol use and HIV risk variables were summarized.

### 3.4. Risk of Bias Assessment

To assess the risk of bias for individual studies, we utilized the Quality Assessment Tool for Observational Cohort and Cross-Sectional Studies developed by the National Heart, Lung, and Blood Institute [17]. The tool includes 14 questions regarding the quality of specific methodological characteristics, including the clarity of the research question, clarity of the study population, sample size and selection, definition and measurement of variables, and statistical analysis methods. Each question may receive a rating of “yes,” “no,” or “not applicable”/“not reported.” The tool is not intended to produce an overall numeric score or tally; therefore, we considered each question individually. M.K. recorded responses to each question for each study, and S.D. checked these responses. There were no conflicting ratings.

### 3.5. Synthesis

We utilized the narrative synthesis process outlined by Popay and colleagues [18], which entails developing a preliminary synthesis, exploring relationships within and between studies, and evaluating the robustness of the synthesis. M.K. and S.D. collaboratively developed the preliminary synthesis by using the data extraction spreadsheet to record findings regarding associations between any of the specified alcohol- and HIV-related risk outcomes. These initial summaries allowed us to explore relationships within and between studies. Specifically, we compared relevant study findings, noted convergent and divergent findings, and identified plausible reasons for variability, such as differences in methodological characteristics across studies. To address the robustness of the synthesis following Popay and colleagues [18], we considered how patterns across the risk of bias assessments, the number of studies included in our review, and variability in study methods potentially impacted the trustworthiness of the overall evidence. We did not conduct sensitivity analysis, as no meta-analysis was performed. All studies identified as eligible for the review were included in the narrative synthesis. We constructed tables to present the methodological characteristics, relevant findings, and risk of bias assessment results for each study. All authors approved the final synthesis. In the Section 4, we provide a narrative summary of these findings.

## 4. Results

### 4.1. Study Selection

**Figure 1:**
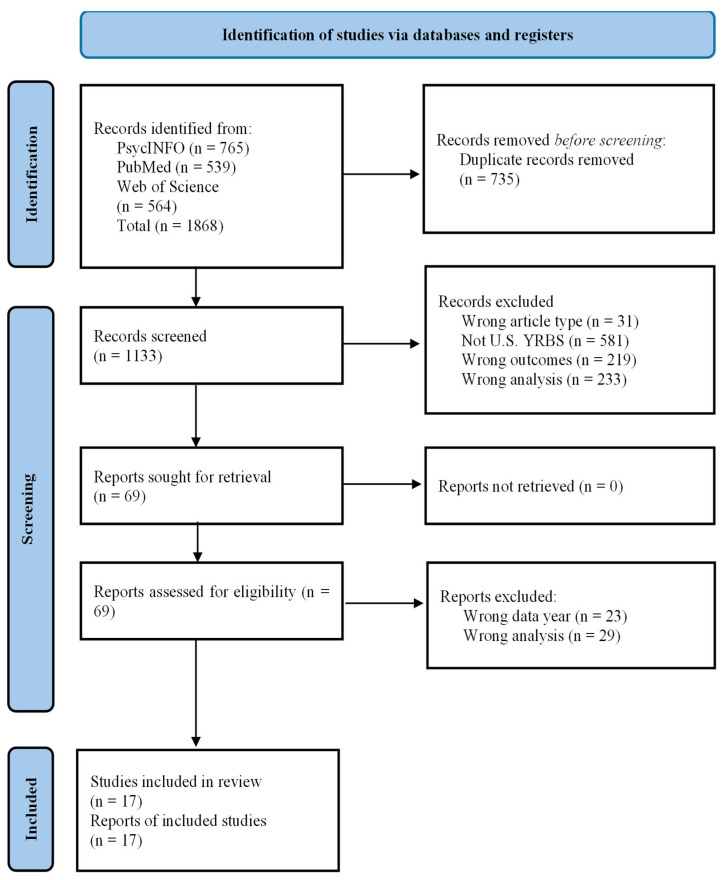
PRISMA Flow Diagram.

Figure 1 displays the results of each phase of the study selection process. The database searches retrieved a total of 1868 records, with 1133 unique records remaining after duplicates were removed. After the title and abstract screening phase, 69 studies met eligibility criteria. The full-text review eliminated 52 studies, leaving 17 for inclusion in the literature review.

### 4.2. Result of Risk of Bias Assessment

Results of evaluations for individual studies using the Quality Assessment Tool for Observational Cohort and Cross-Sectional Studies are displayed in Table S1 of the Supplementary Materials. Certain strengths and limitations of studies relate to the YRBS methodology itself. YRBS data come from large probability samples, and the survey achieved overall participation rates of at least 50% in the examined years [19], minimizing selection and response bias (Q3–Q5 on the assessment tool). YRBS data are also weighted by the CDC, further increasing the representativeness of analyses using this data [19]. Almost all YRBS questions show at least moderate reliability [20], which offers some support for the quality of the measures despite the potential for self-report bias. However, because YRBS data are cross-sectional, all included studies received scores of “no” or “not applicable” to assessment questions related to the ability to detect causal relationships (Q6, Q7, Q10, and Q13).

The characteristics of individual studies also introduced some additional strengths and limitations. All studies accounted for potential confounds through methods including stratifying their analyses (e.g., by sex) and/or controlling for demographic characteristics in their analysis procedures (Q14). In the narrative synthesis, we specify findings that differed between subgroups when analyses were stratified. As a limitation, several studies analyzed exposures with multiple levels (e.g., days of recent alcohol use) as dichotomous variables (Q8; N = 10). The elaboration for the quality assessment tool explains that this dichotomization of ordinal variables loses information about potential dose–response relationships [17]. Another concern included unclear or inconsistent definitions of examined variables (Q9 and Q11; N = 2).

### 4.3. Study Characteristics

Table 1 displays the basic characteristics (i.e., dataset information, sample descriptions, and theoretical frameworks) of each study. Fourteen of 17 (82.4%) studies used data from the national YRBS administration, while one examined data from a single state [21], one used data from a single large urban school district [22], and one pooled data across all states and large urban school districts [23], Additionally, fourteen of seventeen (82.4%) of studies examined only one year of YRBS data, while three (17.6%) pooled data across multiple years [23,24,25].

Studies varied in their target populations; 11 of 17 (64.7%) focused on a general population of high school adolescents, while four (23.5%) restricted their analyses to sexually active adolescents. Two studies (11.8%) focused on demographically specific populations, including high school seniors [25] and American Indian and Alaskan Native students [24]. The analytic sample sizes varied widely depending on the target population, with the smallest including 1178 students [24] and the largest examining a pooled dataset of 541,410 students from state and school district surveys in 2013 and 2015 [23].

### 4.4. Theoretical Frameworks

About half of the studies (9/17; 52.9%) did not mention any theories of youth risk behaviors informing their investigations. Overall, only two studies [28,37] engaged with sociological and psychological theories in detail as conceptual frameworks shaping their hypotheses, study designs, and interpretations of results. Zhao and colleagues [37] framed their study using the Triadic Influence Theory [38], describing how intrapersonal, situational, and cultural factors interactive shape individuals’ behaviors. To explain their hypothesis that associations between substance use and sexual risk behaviors would differ between sexual minority and heterosexual students, Clayton and colleagues [28] discussed the theories that sexual minorities face unique stigma-related stressors that influence their health behaviors (i.e., Minority Stress Theory; [39]) as well as more permissive community norms regarding substance use.

Others referenced theories in describing their study objectives but did not elaborate beyond one or a few sentences. Four studies [26,27,32,36] established their aims of testing relationships between various behavioral and demographic variables by briefly citing Jessor’s Problem Behavior Theory [40], which posits that individual and social environmental factors lead some youth to develop patterns of interrelated risk behaviors. Connell and colleagues [29] mentioned Kandel’s Gateway Model [41] of how different substance use behaviors may develop in succession. Finally, Scroggins and Shacham [35] cited Social Cognitive Theory and Alcohol Myopia and Disinhibition Theories as rationales for examining relationships between alcohol use and sexual risk behaviors. Social Cognitive Theory [42] proposes that behaviors are learned through dynamic interactions between individuals with others in their environments. Alcohol Myopia and Disinhibition Theories [43] suggest that alcohol contributes to risk-taking behaviors by impairing cognitive systems that inhibit impulsive actions. However, Scroggins and Shacham [35] did not define these theories or relate them specifically to the design or interpretation of their study.

### 4.5. Measuring Risk Behaviors

Table 2 summarizes the analysis details and main findings of each study. Of the alcohol-related outcomes included within the scope of our review, studies most frequently examined alcohol use (15/17; 88.2%) and binge drinking (11/17; 64.7%). Several studies examined both separately, and two [24,35] also included a combined variable for overall patterns of alcohol consumption based on alcohol use and binge drinking. Four studies (23.5%) examined age at first drink. For HIV-related outcomes, 12 studies (70.6%) examined number of sexual partners, 12 (70.6%) examined condom use, and 5 (29.4%) considered HIV testing, again with some studies including more than one of these outcomes.

Importantly, the YRBS includes measures of both lifetime and recent (e.g., past 30 days, past 3 months) engagement in many behaviors. Studies in this review varied in their use of lifetime versus recent measures when testing relationships between alcohol and HIV-related behaviors. Of the 15 studies examining alcohol use, nine (60%) considered alcohol use within the past 30 days only, while four studies (26.7%) considered lifetime alcohol use and two (13.3%) used measures of both. Of the 12 studies examining number of sexual partners, seven (58.3%) considered lifetime partners only, two (16.7%) considered number of partners within the past three months [32,33], two (16.7%) used both lifetime and recent measures [24,36] and one (8.3%) did not specify [34].

Studies also varied in their use of binary versus ordinal variables to capture “risk.” In the original YRBS, respondents select from several options for questions about alcohol use, binge drinking, age at first drink, and number of sexual partners. For example, alcohol use in the past 30 days includes seven response options (i.e., 0 days, 1–2, 3–5, 6–9, 10–19, 20–29, all 30), as do questions about number of lifetime and recent sexual partners (i.e., none, 1, 2, 3, 4, 5, 6 or more). Several studies in the review re-coded responses for one or more of these items to binary variables. Of the studies that examined lifetime and/or recent alcohol use, 12 of 15 (80%) used a binary variable reflecting any or no alcohol use. Three studies (20%) maintained ordinal categories of increasing alcohol use frequency [25,29,36]. Similarly, nine of 11 studies (81.8%) examining binge drinking used a binary variable to reflect whether participants had ever binge drank during the past 30 days, while two studies (18.2%) captured multiple levels of binge drinking [29,32]. While Ramisetty-Mikler and Ebama [24] as well as Scroggins and Shacham [35] included binary measures of alcohol use and binge drinking, both also analyzed overall alcohol consumption pattern as having three levels (respectively, did not drink, drank ≥ 3 days but no binge drinking, binge drank ≥ 1 days; and did not drink, drank ≥ 1 days with no binge drinking; binge drank ≥ 1 days). Among the four studies that considered age at first drink, only one re-coded the variable as binary [23], while the remaining others used multiple age categories.

Similarly, studies varied in their use of binary and ordinal variables for HIV-related behavior outcomes. Condom use at last sexual intercourse and ever being tested for HIV were inherently binary outcomes. Of the 12 studies that examined lifetime and/or recent sexual partners, eight (66.7%) re-coded the variable as a binary outcome. One of these studies distinguished between respondents who reported less than two versus two or more sexual partners [33], while the remaining seven distinguished between less than four versus four or more sexual partners. Additionally, one of these studies [30] created a binary, composite “sexual risk behavior” variable to describe respondents who reported both four or more lifetime sexual partners and condom non-use. Four of the 12 studies (33.3%) maintained ordinal categories, with an increasing number of partners representing increased “risk.”

### 4.6. Synthesis of Findings

#### 4.6.1. Number of Sexual Partners and Alcohol-Related Behaviors

Eleven studies examined the relationship between alcohol use and number of sexual partners. As discussed above, studies used varied combinations of lifetime/recent and binary/ordinal measures of both outcomes. In general, studies reported consistent evidence for significant associations between greater alcohol use and greater number of sexual partners [21,25,26,27,28,29,31,36]. However, others revealed nuances within this relationship. Ramisetty-Mikler and Ebama found that respondents who reported recent binge drinking, but not respondents who reported recent drinking with no binge drinking, were significantly more likely to have four or more lifetime sexual partners and two or more recent sexual partners compared to non-drinkers [24]. Mahat and Kelly examined this relationship within groups of respondents who had been tested for HIV and other STDs, finding that recent alcohol use is associated with having four or more sexual partners among those who had tested for other STDs but not HIV [34]. Meanwhile, Desai and colleagues found that reporting any lifetime alcohol use was positively associated with a combined outcome of reporting four or more lifetime sexual partners with condom non-use [30]. Overall, these results indicate that the positive relationship between alcohol use and number of sexual partners is potentially influenced by a complicated array of other factors.

The seven studies examining the relationship between binge drinking and number of sexual partners also generally found evidence for a positive association [21,24,26,27,28,29,32]. Again, studies used a variety of approaches to analyze this relationship. However, there were no studies showing clearly conflicting findings.

Only three studies examined the relationship between age at first drink and number of sexual partners, and all found no significant association between the variables they included [24,25,36]. Specifically, Cavazos-Rehg and colleagues found no relationship between age at first drink and lifetime sexual partners [25], while Ramisetty-Mikler and Ebama as well as Thamotharan and colleagues both found no relationship between age at first drink and lifetime or recent sexual partners [24,36].

#### 4.6.2. Condom Non-Use and Alcohol-Related Behaviors

Evidence for an association between alcohol use and condom use was mixed among the 10 studies examining this relationship. Specifically, three studies consistently indicated that greater alcohol use is associated with greater odds of not using a condom at last sexual intercourse [26,27,33,36]. An additional study [33] supported this relationship only among female respondents. Four studies found no significant association [21,28,34,37]. The remaining two studies considered the role of binge drinking in the relationship between alcohol use and condom use, again with mixed results. Scroggins and Shacham [35] found that respondents who drank without binge drinking had similar odds of condom use to those who did not drink, with both groups being significantly more likely to use condoms than those who binge drank. Ramisetty-Mikler and Ebama [24] did not find this relationship after adjusting for covariates, although at the bivariate level, male respondents who drank without binge drinking were more likely to use condoms compared to those who did not drink at all.

Of the eight studies that tested relationships between binge drinking and condom use, four found consistent evidence that binge drinking is associated with greater odds of not using a condom [26,27,32,35]. Two additional studies reported partial support for this relationship; Clayton and colleagues [28] found that the association between binge drinking and not using a condom was only significant for heterosexual and not lesbian, gay, and bisexual respondents, while Liddon and colleagues [33] found that this relationship was only significant among female and not male respondents. Two studies found no significant relationship between binge drinking and condom use after controlling for demographic and other behavioral variables [21,24]. However, Ramisetty-Mikler and Ebama reported that in bivariate analyses, condom use was significantly lower among male but not female respondents who reported binge drinking versus those who did not drink [24].

Only two studies examined age at first drink in relation to condom use, and neither found a significant association [24,36]. Overall, binge drinking appears to be most consistently associated with not using a condom, while the evidence for alcohol use alone is mixed and age at first drink is less explored.

#### 4.6.3. HIV Testing and Alcohol-Related Behaviors

Few studies explored alcohol-related behaviors in relation to HIV testing status, and findings were highly mixed. Two studies examined the association between alcohol use and HIV testing. Of these, Mahat and Kelly reported that recent alcohol use was not associated with ever being tested for HIV [34], while Thamotharan and colleagues reported that both lifetime and recent alcohol use were associated with having been tested for HIV [36]. Two studies also examined binge drinking in relation to HIV testing. Gao et al. (2017) found no relationship [22]. Outlaw and colleagues found that binge drinking was associated with increased odds of HIV testing for female students only, but this relationship was not significant after controlling for other substance use [23]. Finally, only Outlaw et al. (2022) tested whether age at first drink was related to HIV testing; they found that drinking alcohol before age 13 was associated with increased odds of HIV testing among male students only, but this relationship again lost significance after controlling for other substance use [23]. Based on the studies in this review, alcohol-related behaviors alone may not be reliable indicators of HIV testing status.

## 5. Discussion

This systematic review aimed to synthesize the existing literature examining the relationships between alcohol-related behaviors (alcohol use, binge drinking, and age of first alcohol use) and HIV-related risk behaviors (number of sexual partners, condom non-use, and HIV testing) among adolescents, drawing from studies that utilized YRBS data. Our findings largely support the notion that alcohol use, and particularly binge drinking, is significantly associated with a greater number of sexual partners. However, the evidence is more mixed regarding the association between alcohol use and condom non-use, and there is limited data suggesting that age of first alcohol use is not associated with condom use. Furthermore, with less attention paid to the relationships between alcohol-related variables and HIV testing, the available evidence does not consistently suggest a clear relationship.

### 5.1. Nuances in Findings and Methodological Considerations

The mixed results observed across studies likely reflect significant variability in their methodological approaches. As our results highlight, studies diverged in their use of lifetime versus recent measures for both alcohol-related and HIV-related behaviors. For instance, among studies examining alcohol use, 60% focused on past 30-day use, while others considered lifetime use or both. Similarly, for number of sexual partners, measures ranged from lifetime to past three months. This temporal discrepancy in measurement can lead to differing conclusions, as the immediate effects of recent alcohol consumption on sexual behavior might differ from the cumulative impact of lifetime drinking habits.

Another key factor contributing to varied findings is the analytical approach to ordinal variables. The YRBS provides several response options for behaviors like alcohol use frequency, binge drinking frequency, and number of sexual partners. However, our review found that a substantial majority of studies (80% for alcohol use and 81.8% for binge drinking) chose to dichotomize these variables into binary outcomes (e.g., any alcohol use vs. no alcohol use, ever binge drank vs. never). While simplifying analysis, this practice leads to a loss of valuable information and can obscure nuanced relationships. For example, a respondent who drinks 1–2 days a month is grouped with someone who drinks 20–29 days a month, masking potential differences in risk profiles.

Evidence from some studies suggests that considering more than two levels of “risk” can reveal patterns that would not be apparent otherwise. Ramisetty-Mikler and Ebama as well as Scroggins and Shacham both differentiated between respondents who did not drink, drank without binge drinking, and binge drank [24,35]. Both studies found evidence that for some HIV-related behaviors, binge drinking but not non-binge drinking was associated with greater risk [24,35]. Similarly, Cavazos-Rehg and colleagues found that greater frequency of lifetime alcohol use was associated with a greater number of lifetime sexual partners following a stepwise pattern [25], indicating that increasing levels of alcohol consumption correspond to increasing numbers of partners. These findings underscore the importance of maintaining the ordinality of YRBS variables where appropriate to capture the full spectrum of risk and identify specific thresholds or patterns of behavior that are most impactful. Furthermore, variability in the control variables included in multivariate analyses could contribute to the heterogeneity of findings. Our risk of bias assessment noted that some studies did not clearly report how potential confounds were controlled, which can influence the observed associations between alcohol and HIV-related behaviors.

### 5.2. Consistency and Gaps in Evidence

Despite the methodological variations, our review identified generally consistent evidence that alcohol use, and especially binge drinking, but not age of first alcohol use, was associated with a greater number of sexual partners. This association suggests a link between current or recent problematic alcohol consumption and increased sexual risk-taking, aligning with disinhibition theories where alcohol impairs judgment and reduces inhibitions. Conversely, the minimal and often non-significant findings concerning age of first alcohol use in relation to number of sexual partners or condom use indicate that the initiation of drinking might be less directly linked to these specific HIV risk behaviors than ongoing or heavy consumption patterns.

For condom use, the evidence was notably mixed. While some studies showed that greater alcohol use or binge drinking correlated with greater odds of condom non-use, others found no significant association. This inconsistency might be partly attributed to methodological differences, but it could also reflect a more complex interplay of factors influencing condom use decisions that are not solely dictated by alcohol consumption. Indeed, a wide range of psychosocial factors across multiple levels of analysis (e.g., personality traits, partner attitudes, perceived cultural norms) are associated with condom use decisions in the wider literature on adolescent sexual behaviors [44]. The limited evidence indicating no relationship between age of first alcohol use and condom use further emphasizes that early initiation alone may not be a strong determinant of later condom use practices.

Regarding HIV testing status, our review found too little evidence to suggest any consistent relationships between alcohol-related variables and HIV testing status. The few studies that explored this link yielded mixed results, with some reporting no association and others reporting varied associations that lost significance after controlling for other substance use. This highlights a critical gap in the existing YRBS-based literature and suggests that alcohol use alone may not be a reliable indicator of HIV testing history among adolescents. Other factors, such as perceived risk, access to testing services, or broader health-seeking behaviors, might be more influential.

### 5.3. Theoretical Frameworks and Future Directions

A significant finding from our analysis was that only about half of the included studies cited any theoretical frameworks informing their investigations. Specifically, Problem Behavior Theory, Triadic Influence Theory, Minority Stress Theory, Permissive Norms Theory, Social Cognitive Theory, Alcohol Myopia Models, and Disinhibition Theory were cited by a minority of studies. However, these instances of engagement mostly involved brief references rather than a detailed discussion or analytical application. As others have argued [45], integrated sociological and psychological theories are needed to elucidate mechanisms driving adolescent risk behaviors within wider social contexts.

Theoretically informed analyses could bring clarity to areas of research currently characterized by mixed findings. For example, experimental literature demonstrates that alcohol consumption leads to greater intentions for unprotected sex when participants are exposed to sexual cues, consistent with Alcohol Myopia Theory’s predictions about the disinhibitory effects of alcohol [3]. If alcohol myopia follows a dose–response trend, this may help explain why binge drinking relates to condom non-use and a greater number of sexual partners more consistently than alcohol use in general. Studies that differentiate between more or less intense patterns of alcohol consumption (e.g., binge drinking versus non-binge drinking at various frequencies) could explore this possibility.

Furthermore, theories that account for the interactions between individual risk and resilience factors, immediate social environments, and broader structural and cultural contexts, including but not limited to Triadic Influence Theory, are necessary to identify for whom and in what circumstances relationships between alcohol use and HIV risk behaviors are more salient. YRBS includes several specific variables that can support these complex analyses, including demographic information and mental health; interpersonal experiences of violence and bullying; and structural factors such as school and neighborhood safety, access to sexual health education, and socioeconomic circumstances [19]. As one application, U.S. adults [46] and youth [47] living in communities with fewer economic and social support resources experience disproportionate HIV vulnerability; future YRBS research could examine how access to essential resources might influence alcohol- and HIV-related behaviors among youth, separately and in relation to one another.

YRBS data can also be integrated with other datasets, for example, on state policies. Studies might examine whether the structural factor of sexual health education, which varies in content and availability by state, intervenes in relationships between alcohol- and HIV-related behaviors. Existing YRBS research shows that adolescents in states with more comprehensive sex education policies have a lower prevalence of unprotected sex [48]. State curricula that include information about both sexual health and substance use may also be an important but unevenly distributed resource. By explicitly drawing on and testing theoretical propositions, future research can clarify the underlying mechanisms and pathways through which alcohol-related behaviors influence HIV-related risk behaviors in youth. This would allow for more targeted and effective prevention and intervention strategies at both individual and structural levels, which are greatly needed [49].

The heterogeneity of findings highlighted in this review—particularly regarding the relationship between alcohol use and condom use and HIV testing—underscores a clear need for future research to move beyond simple correlational analyses [50]. Future studies should intentionally explore whether key methodological differences contribute to divergent findings. Specifically, research should compare how results differ when using dichotomous versus ordinal measures, or when analyzing data from different collection years. Furthermore, future studies should also perform robust subgroup analyses, stratifying their findings by sex, sexual orientation, and ethnicity to determine if and how these associations differ across populations. A more standardized approach to the operationalization of variables and an explicit focus on exploring heterogeneity will be essential for building a more cohesive body of evidence and, ultimately, for informing future meta-analyses on this topic.

Finally, while the YRBS provides valuable large-scale data, its cross-sectional nature limits the ability to establish causal relationships. Future research, perhaps integrating longitudinal data or employing advanced statistical methods that account for temporal dynamics, would be crucial to better understand the causal pathways between alcohol consumption and HIV risk behaviors among adolescents.

### 5.4. Limitations of the Studies in This Review

The literature synthesized in this review includes several limitations imposed by the characteristics of the source data and the methodological approaches of the included studies. As our risk of bias assessment underscored, the cross-sectional nature of YRBS data precludes the establishment of causal relationships. Consequently, while we discern consistent associations between specific alcohol-related behaviors and HIV risk behaviors, asserting that one directly causes the other remains beyond the scope of this review. Future inquiries necessitate longitudinal designs or advanced causal inference methods to untangle these temporal and directional complexities.

Further limiting our ability to draw definitive conclusions is the considerable methodological heterogeneity in variable operationalization and measurement across studies. Our findings detailed divergent practices in using lifetime versus recent measures for both alcohol consumption and number of sexual partners. This divergence is consequential because lifetime and recent measures of one behavior may relate differently to another outcome. However, too few studies in our review used both measures where available to assess this possibility in a controlled manner. The temporal dimensions of other risk behaviors are also impossible to analyze using available YRBS data. YRBS only asks whether respondents have ever been tested for HIV in their life, precluding examinations of factors associated with regular testing behavior. Likewise, YRBS exclusively measures respondents’ condom use at their last sexual encounter, which may not be an accurate indicator of general condom use.

The widespread dichotomization of ordinal YRBS variables (e.g., collapsing frequency scales into binary “any vs. none” categories for alcohol use and number of sexual partners) resulted in a significant loss of detail. This analytical simplification can obscure nuanced patterns of risk not apparent in binary analyses, such as dose–response relationships. For example, adolescents who consume alcohol very frequently may experience different social situations and vulnerabilities influencing their sexual health behaviors than adolescents who consume alcohol once or twice per month, but binary analyses of alcohol use conflate these groups. Moreover, measurement variability among studies complicates the synthesis of findings and may contribute to the mixed evidence observed, particularly regarding relationships between alcohol and condom use. Future research can preserve the specificity of YRBS variables with several possible levels by utilizing methods such as ordinal regression models [51].

Constraints of the YRBS instrument itself also impose limitations. While providing robust, large-scale self-reported behavioral data, the YRBS does not capture the full spectrum of psychosocial, interpersonal, or environmental factors that influence adolescent risk-taking. Critical variables such as the social contexts surrounding alcohol use, intricate peer dynamics, or detailed individual psychological states that might mediate the alcohol-HIV risk behavior link are largely unmeasured. The omission of these potential confounders or mediating variables could significantly influence the observed associations, presenting an incomplete picture.

The finding that most included studies were atheoretical is a significant limitation of the existing literature and presents a key opportunity for future research. Research on adolescent risk behaviors often examines associations between variables without explicitly testing theoretically grounded hypotheses [52]. This approach, while valuable, may oversimplify the complex interplay of individual behaviors and their social context. We argue that future studies should explicitly integrate and test theoretical frameworks to interpret how and why these behaviors are related. For instance, Social Cognitive Theory [53] could be applied to explore how social and environmental factors—such as peer influence or community norms—interact with individual self-efficacy to shape decisions around alcohol use and sexual behaviors. Furthermore, Alcohol Myopia and Disinhibition Theories could be used to directly test whether alcohol consumption narrows an individual’s focus to immediate cues, reducing inhibitions and making them more likely to engage in risky sexual behaviors. The consistent finding in our review that binge drinking, more so than alcohol use in general, is associated with a greater number of sexual partners is a finding that aligns with these theories’ predictions about the disinhibitory effects of more intense alcohol consumption. Finally, theories that account for the interactions between individual risk and resilience factors, immediate social environments, and broader structural and cultural contexts, such as Triadic Influence Theory [54], are necessary to identify for whom and in what circumstances relationships between alcohol use and HIV risk behaviors are more salient. By applying such frameworks, researchers can move beyond simply documenting associations to developing a deeper, multi-level understanding of the mechanisms that link alcohol use to HIV-related risk behaviors.

Finally, a notable overall limitation of the reviewed literature is the limited and often superficial engagement with theoretical frameworks. Over half of the studies included cited no theory, while many of the remainder offered only a brief, unelaborated mention. This theoretical vacuum frequently translates into analyses focused primarily on identifying statistical associations rather than elucidating the specific pathways through which alcohol influences HIV risk behaviors. Such an approach impedes the development of robust explanatory models and the design of theoretically informed, targeted interventions. The scarcity of studies investigating HIV testing in relation to alcohol-related behaviors further underscores a critical research gap, curtailing our capacity to draw meaningful conclusions on this vital public health outcome.

### 5.5. Limitations of This Review

Despite adhering to rigorous systematic review methodologies, this review possesses certain limitations that warrant consideration. Firstly, our search strategy, while comprehensive across three major databases (PubMed, Web of Science, and PsycINFO), was restricted to peer-reviewed journal articles published in English. This restriction means that relevant studies published in other languages or in grey literature (e.g., dissertations, government reports, conference proceedings) were excluded, potentially introducing a language and publication bias. While we aimed for broad coverage, the omission of these sources could mean our synthesis does not encompass the entire available evidence base.

Secondly, the scope of our review was limited to YRBS data from 2005–2021. While this provided a focused analysis of a specific and highly utilized dataset, it means our findings may not be generalizable to research utilizing other adolescent health surveys or data collected outside this timeframe. Our findings are also restricted to the United States, although an international meta-analysis found that alcohol consumption is significantly associated with inconsistent condom use and having multiple sexual partners [55]. Addressing alcohol use, sexual health concerns, and their intersections among adolescents remains a global health priority [56].

A further limitation is the review’s exclusive focus on HIV-related risk behaviors. Although the YRBS data includes some information on other sexually transmitted infections (i.e., During the past 12 months, have you been tested for a sexually transmitted disease (STD) other than HIV, such as chlamydia or gonorrhea?), and a robust body of research links alcohol to their acquisition [57], our synthesis was restricted to a subset of variables directly tied to HIV. This purposeful scoping decision means our findings may not be fully generalizable to the broader public health concern of STIs among adolescents.

Finally, due to the substantial heterogeneity in study designs, variable operationalizations (e.g., lifetime vs. recent measures, dichotomized vs. ordinal variables), and statistical analyses across the included primary studies, we were unable to perform a quantitative meta-analysis. Our synthesis instead relied on a narrative approach. While this method enables the identification of patterns, nuances, and challenges within the literature, it prevents the generation of precise aggregate effect estimates and the statistical assessment of overall effect sizes, which a meta-analysis could provide.

## 6. Conclusions

This systematic review provides a comprehensive synthesis of the literature examining the relationships between alcohol-related behaviors and HIV risk behaviors among adolescents, drawing from studies utilizing YRBS data from 2005 to 2021. Our findings indicate an overall link between problematic alcohol consumption and some forms of increased sexual risk-taking among youth, although further research is needed to address certain gaps and inconsistencies in the literature. Mixed findings may stem from differences in studies’ measurement and analysis procedures, highlighting a need for more theoretically engaged research that considers multiple dimensions of health behaviors (e.g., intensity, frequency, timing, social context). The findings of our review are also limited by the cross-sectional nature of YRBS data, preventing causal conclusions.

Future research can clarify areas of uncertainty by maintaining the granularity of YRBS data, explicitly integrating and testing theoretically informed hypotheses, and exploring longitudinal designs to elucidate causal pathways. Changes to YRBS could further support researchers in conducting more complex analyses. For example, rather than asking whether respondents used a condom at last sexual intercourse or whether they have ever been tested for HIV, measuring respondents’ frequency of condom use and HIV testing over a defined period would enable more robust inferences about how *patterns* of these behaviors relate to patterns of alcohol use. Questions about the immediate social context of alcohol use (e.g., at a party, with a partner, alone) and respondents’ motivations for alcohol use (e.g., enjoyment, to meet social expectations, emotional coping) would also allow researchers to investigate how different psychological and social circumstances influence behavioral patterns.

Despite some ambiguous findings, this review underscores the importance of addressing alcohol use, especially binge drinking, as a potential factor in adolescent sexual health. Continued attention to this intersection is crucial for developing targeted, effective, and evidence-based interventions aimed at mitigating risk behaviors among young people. For health professionals and educators, our results indicate that efforts to support adolescents’ sexual health should include conversations about alcohol consumption in the context of sexual relationships and decision-making. This echoes suggestions from the CDC that sexual health education should incorporate information on substance use [58]. Likewise, professionals working with youth who use alcohol should ensure that these youth have access to sexual health resources, such as HIV testing, condoms, and educational materials [59]. Schools and community organizations can use existing programs such as the CDC’s Teens Linked to Care program [56] as blueprints for connecting adolescents to professional and peer support around both alcohol use and sexual health concerns. Given this review’s findings regarding associations between binge drinking and HIV risk behaviors, approaches focused on reducing more intense patterns of alcohol use may be particularly useful.

The production and application of research on adolescent health behaviors ultimately depends on effective policies. First, ensuring that public health surveys like YRBS continue collecting data on sex, gender, sexual orientation, race, ethnicity, and socioeconomic status should be a priority. Studies in this review paid limited attention to how relationships between alcohol use and HIV risk behaviors may differ across social locations, beyond sex-stratified analyses. However, sexual minority youth continue to face disparities in both alcohol use and HIV risk behaviors [28], and evidence beyond this review demonstrates that sexual minority and racially marginalized youth report lower access to certain sexual health resources [57]. Researchers must have the capacity to track such disparities to promote the health of all adolescents. Relatedly, policies from the school to state levels can invest in and protect comprehensive health education and services, addressing adolescents’ health behaviors in the context of their diverse identities, resources, relationships, and environments.

## Figures and Tables

**Table 1:** Basic Study Characteristics.

Author (Year)	Study Aims and Theoretical Frameworks	YRBS Datasets and Target Population	Sample Details
Ahmadi-Montecalvo et al. (2019) [26]	Examine the co-occurrence of multiple health-risk behaviors to determine whether there are any differences in the pattern of co-occurrence by sex. Theories: Problem behavior theory.	Level: National Year(s): 2013 Population: High school students; analyzed female and male subgroups	Criteria: None specified Size: 13,571 (6950 male, 6621 female) Age range: <12–18
Aslam et al. (2023) [27]	Ascertain inconsistencies in the trend of co-occurrence among different minority and majority races by sex of teenage health risk behavior patterns such as smoking, behaviors contributing to deliberate and unintentional injuries, risky sexual behavior, and sedentary lifestyle. Theories: Problem behavior theory.	Level: National Year(s): 2013 Population: High school students; analyzed female and male subgroups	Criteria: None specified Size: 13,583 (male and female subsample sizes NR) Age range: <12–18
Cavazos-Rehg et al. (2011) [25]	Examine how age of substance use initiation and variations in use (i.e., experimental/new user, moderate, heavy versus non-user) are associated with increased number of sexual partners. Theories: None.	Level: National Years: 1999–2007 (pooled) Population: High school seniors; analyzed female and male subgroups	Criteria: included 12th graders aged 17 and older; excluded racial/ethnic groups other than White, African American, and Hispanic; excluded those with missing data Size: 11,268 Age range: ≥17
Clayton et al. (2019) [28]	Explore potential differences in associations between sexual risk taking and substance use by sexual identity. Theories: Minority Stress theory, permissive norms.	Level: National Year(s): 2015 Population: High school students; compared lesbian, gay, and bisexual and heterosexual students	Criteria: Excluded respondents who were “not sure” of their sexual identity Size: 14,200 (12,954 heterosexual; 1246 lesbian, gay, and bisexual) Age range: 12–19
Connell et al. (2009) [29]	Assess the co-occurrence of patterns of adolescent substance use and sexual behavior and test for potential moderating effects of gender. Theories: Gateway hypothesis.	Level: National Year(s): 2005 Population: High school students; analyzed female and male subgroups	Criteria: None specified Size: 13,953 Age range: ≤14–≥18
Desai et al. (2021) [30]	Identify the prevalence of sexual risk behaviors in U.S. high school students and study the difference in the odds of sexual risk behaviors for various substances. Theories: None.	Level: National Year(s): 2019 Population: High school students	Criteria: Excluded respondents with missing data for age, sex, race, grade, and/or substance use Size: 11,191 Age range: ≤14–≥18
Finch & Pierson (2011) [31]	Identify typologies of adolescents based on their propensity for engaging in sexually and substance use risky behaviors. Theories: None.	Level: National Year(s): 2009 Population: High school students	Criteria: None specified Size: 16,410 Age range = NR, *M* = 16.03, *SD* = 1.23
Gao et al. (2017) [22]	Estimate the prevalence of and identify risk factors for not receiving an HIV test among New York City adolescents with a history of sexual intercourse. Theories: None.	Level: Large urban school district (New York City) Year(s): 2013 Population: Sexually experienced New York City high school students	Criteria: Included respondents who replied “yes” or “no” to ever receiving HIV testing and reported lifetime sexual intercourse; excluded respondents with missing data for relevant characteristics and risk factors Size: 1199 Age range: ≤14–≥18
Hingson & Zha (2018) [32]	Explore whether adolescent binge drinking at ≥ twice versus < twice the age-/gender-specific thresholds versus non-binge drinking heightens associations of drinking with health-risk behaviors. Theories: Problem behavior theory.	Level: National Year(s): 2015 Population: High school students	Criteria: Excluded respondents younger than 14 Size: 13,191 Age range: 14–≥18
Liddon et al. (2016) [33]	Describe the prevalence of withdrawal as their primary method of pregnancy prevention at last sexual intercourse among sexually active US high school students and associations with sexual risk and substance use. Theories: None.	Level: National Year(s): 2011 Population: Sexually active high school students; analyzed female and male subgroups	Criteria: Included respondents who reported current sexual activity (i.e., past three months); excluded respondents who answered “I did not have sexual intercourse” to either of two contraceptive questions Size: 4793 Age range: NR
Mahat & Kelly (2023) [34]	Identify potential contributors to high-risk sexual behaviors among sexually active adolescents who were tested for HIV and STDs compared to those who did not test for HIV and STDs. Theories: None.	Level: National Year(s) 2019 Population: Sexually active high school students; compared those who had vs. had not been tested for HIV and STDs	Criteria: Included respondents who reported current sexual activity Size: 3226 (1010 HIV tested, 2216 not HIV tested; 1353 STD tested, 1873 not HIV tested) Age range = NR, *M* = 16.4, *SD* = 1.2
McGuire et al. (2012) [21]	Describe the patterns of substance use, sexual risk behaviors, and mental health problems and examine the relationship between substance use and sexual risk behaviors. Theories: None.	Level: State (Mississippi) Year(s): 2009 Population: Public high school students in Mississippi	Criteria: Excluded respondents who did not report their race/ethnicity as non-Hispanic White or non-Hispanic Black Size: 1789 Age range: ≤14–≥18
Outlaw et al. (2022) [23]	Examine high school student characteristics and substance use in relation to self-reported HIV testing. Theories: None.	Level: All states and large urban school districts (pooled) Year(s): 2013–2015 (pooled) Population: High school students; analyzed female and male subgroups	Criteria: Excluded respondents with missing data for race/ethnicity, sex, age, sexual behavior, and/or HIV testing Size: 541,410 Age range: ≤14–≥18
Ramisetty-Mikler & Ebama (2011) [24]	Investigate the patterns of alcohol, other drug use, and sexual risk behavior among homogenous and biracial American Indian and Alaska Native youth; analyze the association between alcohol/drug use and sexual risk. Theories: None.	Level: National Year(s): 2005–2007 (pooled) Population: American Indian and Alaska Native high school students; analyzed female and male subgroups	Criteria: Included respondents who identified as American Indian and Alaska Native, either alone or with White, African American, or Hispanic race/ethnicity; excluded those who identified as American Indian and Alaska Native and multiple other races Size: 1178 Age range: ≤14–≥17
Scroggins & Shacham (2021) [35]	Compare the associations between alcohol-use patterns and condom utilization among US adolescents to more accurately determine risk of STIs. Theories: Social cognitive theory, alcohol myopia models, disinhibition theory.	Level: National Year(s): 2017 Population: Sexually active high school students	Criteria: Limited to students who reported ≥1 sexual partner in the past three months Size: 3732 Age range: NR
Thamotharan et al. (2015) [36]	Examine the relationship between substance use and high-risk sexual behavior. Theories: Problem behavior theory.	Level: National Year(s): 2011 Population: High school students	Criteria: None specified Size: 15,425 Age range: ≤12–≥18
Zhao et al. (2017) [37]	Provide information about the differences in condomless sex between male and female adolescents, including multiple sex partners and substance use. None: Triadic influence.	Level: National Year(s): 2011 Population: Sexually active high school students; analyzed female and male subgroups	Criteria: Excluded respondents who did not have sexual intercourse during the previous three months and/or did not report their gender, grade, or condom use at last sexual intercourse. Size: 4968 Age range: ≤12–≥18

Note. HIV = human immunodeficiency virus. STD = sexually transmitted disease. STI = sexually transmitted infection.

**Table 2:** Summary of Analyses and Findings.

Authors (Year)	Alcohol and HIV Risk Variables	Definition of Risk Behaviors and Analysis Methods	Relevant Findings
Ahmadi-Montecalvo et al. (2019) [26]	Alcohol: Alcohol use in past 30 days (≥1 vs. 0 days); binge drinking (≥5 drinks in a row) in past 30 days (≥1 vs. 0 days) HIV: Lifetime sexual partners (<4 vs. ≥4 people); condom use during last sexual intercourse (yes, no)	Definition: Examined 40 “health risk behaviors” in total; analyzed all as binary variables Analysis: Latent class analysis including 40 health risk behaviors, stratified by sex	Among male, female, and all respondents, the class with high levels of alcohol use had a greater probability of ≥ 4 lifetime sexual partners and condom non-use compared to the class with a low prevalence of all examined behaviors.
Aslam et al. (2023) [27]	Alcohol: Alcohol use in past 30 days (≥1 vs. 0 days); binge drinking (≥5 drinks in a row) in past 30 days (≥1 vs. 0 days) HIV: Lifetime sexual partners (<4 vs. ≥4 people); condom use during last sexual intercourse (yes, no)	Definition: Examined 40 “health risk behaviors” in total; analyzed all as binary variables Analysis: Latent class analysis including 40 health risk behaviors, stratified by sex	For both male and female respondents, the analysis identified a class characterized by a high co-occurrence of alcohol with multiple sexual partners and condom non-use.
Cavazos-Rehg et al. (2011) [25]	Alcohol: Age at first drink (≤12, 13–14, ≥15); alcohol use intensity (non-users, experimental/new users [1–9 lifetime uses], moderate users [10–99 lifetime uses], heavy users [100+ lifetime uses]) HIV: Lifetime sexual partners (0, 1, 2 … 5, ≥6).	Definition: Used categories of increasing risk (younger age at first drink, heavier alcohol use, more lifetime sexual partners) Analysis: Multinomial multivariable logistic regression analyses with alcohol use variables as predictors of HIV risk behaviors, stratified by sex	For both female and male groups, greater intensity of alcohol use was associated with greater odds of a higher number of sexual partners, following a stepwise pattern. Age at first drink of alcohol had little effect on number of sexual partners.
Clayton et al. (2019) [28]	Alcohol: Alcohol use in past 30 days (≥1 vs. 0 days); binge drinking (≥5 drinks in a row) in past 30 days (≥1 vs. 0 days) HIV: Lifetime sexual partners (<4 vs. ≥4 people); Condom use at last sexual intercourse (yes vs. no)	Definition: Described variables as “substance use and risky sexual behaviors”; analyzed all as binary variables Analysis: Logistic regression models with alcohol use variables as predictors of HIV risk behaviors, stratified by sexual identity	Among both heterosexual and lesbian, gay, and bisexual groups, alcohol use and binge drinking were both associated with having ≥ 4 lifetime sexual partners. Alcohol use was not associated with condom non-use for either group. Binge drinking correlated with condom non-use in heterosexuals.
Connell et al. (2009) [29]	Alcohol: Alcohol use in past 30 days (no history, no past-month use, infrequent [1–5 days], frequent [≥6 days]); binge drinking in past 30 days (no history, no past-month use, infrequent [1–5 days], frequent [≥6 days]) HIV: Sexual partners in past 3 months and lifetime (0, 1, 2…5, ≥6); condom use during last sexual intercourse (no history, yes, no); history of HIV testing (yes, no, don’t know)	Definition: Described variables as “substance use and sexual risk behaviors”; analyzed all as ordinal variables Analysis: Latent class analysis, with multinomial logistic regression to examine substance use class as predictor of sexual behavior class; also tested interaction effects between gender and substance use on sexual behavior.	For the class characterized by alcohol use alone versus nonusers, odds of belonging to the class with multiple lifetime but not recent sexual partners were significantly greater, and odds of being in the class with multiple lifetime and recent sexual partners were also significantly greater. These patterns were stronger for female versus male respondents.
Desai et al. (2021) [30]	Alcohol: Lifetime alcohol use (yes, no; coded from question about age of initiation) HIV: Lifetime sexual partners (<4 vs. ≥4 people); condom use at last sexual intercourse (yes, no)	Definition: Described “substance use and sexual risk behaviors”; binary defined “sexual risk behaviors” as reporting both ≥ 4 lifetime sexual partners and condom non-use Analysis: Multivariable logistic regression analysis to examine various substance use outcomes as predictors of odds of sexual risk behaviors	After adjusting for age, sex, race, grade and other substance use, the odds of sexual risk behaviors were significantly higher for participants who had ever used alcohol.
Finch & Pierson (2011) [31]	Alcohol: Alcohol use in past 30 days (≥1 vs. 0 days) HIV: Lifetime sexual partners (<4 vs. ≥4 people)	Described all variables as “risky sexual and substance use behaviors”; analyzed as binary Mixed item response theory analysis; multivariate analysis of variance and discriminant analysis comparing the latent classes on the sums of risky sexual and substance use behaviors engaged in.	The class with the greatest prevalence of risky sexual behaviors (Class 1) had a greater prevalence of alcohol use compared to other classes. Other classes were defined by prevalent alcohol use but not sexual risk behaviors (Class 2), prevalent sexual risk behaviors but not alcohol use (Class 3), and a low prevalence of all risk behaviors (Class 4).
Gao et al. (2017) [22]	Alcohol: Binge drinking (≥5 drinks in a row) in past 30 days (≥1 vs. 0 days) HIV: History of HIV testing (yes, no)	Definition: Considered binge drinking and other substance use as risk factors; analyzed as binary Analysis: Modified Poisson regression to examine alcohol and other substance use in relation to HIV testing.	No significant association was found between binge drinking and HIV testing.
Hingson & Zha (2018) [32]	Alcohol: Binge drinking in past 30 days (abstained from alcohol, drank but did not binge, binged < twice the age- and sex-specific threshold, or binged ≥ twice the threshold). HIV: Sexual partners in past 3 months (0, 1, 2, ≥3); condom use at last sexual intercourse (yes, no)	Definition: Described all variables as “health risk behaviors”; analyzed alcohol variables and number of sexual partners as ordinal Analysis: Logistic regression analyses with pairwise comparisons of number of sexual partners by different drinking levels	Compared to non-drinkers, those binging ≥ twice thresholds had significantly greater odds of multiple sexual partners in the past 3 months and significantly lower odds of condom use. Those binging ≥ twice thresholds versus < twice thresholds and non-binge drinkers were significantly more likely to have multiple sexual partners. The same held for binging at < twice thresholds compared to non-binge drinkers.
Liddon et al. (2016) [33]	Alcohol: Alcohol use in past 30 days (≥1 vs. 0 days); binge drinking (≥5 drinks in a row) in past 30 days (≥1 vs. 0 days) HIV: Sexual partners in past 3 months (<2 vs. ≥2 people) (only as a predictor of condom use, not a separate outcome); condom use (yes, no) and other contraceptive use	Definition: Described variables as “substance use and sexual risk” behaviors; analyzed alcohol measures as binary and condom/contraceptive use as ordinal (no method, condoms alone, highly effective contraceptive method) Analysis: Chi-square analyses; bivariate and multivariable logistic regression analyses with alcohol variables and multiple sexual partners as predictors of condom and contraceptive use.	The prevalence of alcohol use and binge drinking was highest among students who did not use condoms or other contraceptives, lower among those who used condoms alone, and lowest among students who used a highly effective method. This pattern held among female but not male students.
Mahat & Kelly (2023) [34]	Alcohol: Alcohol use in past 30 days (≥1 vs. 0 days) HIV: Sexual partners (did not specify lifetime or recent; <4 vs. ≥4 people); condom use at last intercourse (yes, no); ever being tested for HIV (yes, no)	Definition: Considered all variables as “risk-taking behaviors”; analyzed all variables as binary Analysis: Chi-square analyses to test differences between testing status groups; logistic regression to examine alcohol use in relation to sexual risk behaviors	Those who were never tested for HIV were more likely to use condoms and less likely to have multiple sexual partners than those tested for HIV, with a similar pattern for STD testing. No significant differences were found by HIV or STD testing status for alcohol use. Among those tested for HIV, alcohol use did not correlate with condom use or multiple sexual partners. Among those tested for STDs, alcohol use correlated with multiple sexual partners but not condom use.
McGuire et al. (2012) [21]	Alcohol: Lifetime alcohol use (≥1 vs. 0 days); binge drinking (≥5 drinks in a row) in past 30 days (≥1 vs. 0 days) HIV: Lifetime sexual partners (<4 vs. ≥4 people); condom use at last intercourse (yes, no)	Definition: Described variables as “substance use and sexual risk behaviors”; analyzed all variables as binary Analysis: Chi-squared test to examine sex differences in risk behaviors; bivariate and multiple logistic regression models to examine alcohol variables in relation to sexual risk behaviors	In multivariate analyses, ever drinking alcohol and binge drinking were both positively associated with having four or more lifetime sexual partners. Alcohol use and binge drinking were not associated with condom use.
Outlaw et al. (2022) [23]	Alcohol: Lifetime alcohol use (≥1 vs. 0 days); age of first drink of alcohol (<13 vs. ≥13 years); binge drinking (≥5 drinks in a row) in past 30 days (≥1 vs. 0 days) HIV: ever being tested for HIV (yes, no)	Definition: Described all variables as behaviors associated with risk for HIV; analyzed all variables as binary Analysis: Sex-stratified, stepwise multivariable logistic models to examine associations of student characteristics and substance use with odds of ever having received a HIV test	When controlling for student characteristics and alcohol use, binge drinking correlated with increased odds of HIV testing for female students only, while alcohol use before age 13 correlated with increased odds of testing for male students only. After controlling for drug use, these relationships were not significant.
Ramisetty-Mikler & Ebama (2011) [24]	Alcohol: Lifetime alcohol use (≥1 vs. 0 days); age at first drink (≤10, 11–12, 13–14, and ≥15 years); episodic drinking, based on frequency and amount of drinking in past 30 days (never drank, drank ≥ 3 days but no binge drinking, binge drank ≥ 1 days) HIV: Lifetime and past 3-month sexual partners (never had sex, 1 partner, ≥2 partners); condom use at last intercourse (yes, no)	Definition: Referred to “alcohol/drug use predictors of sexual risk taking”; examined alcohol use as binary and other multilevel variables as ordinal Analysis: Sex-specific bivariate and multivariate analyses to identify associations between alcohol and other drug use and sexual behaviors; individual logistic regression and multinomial models to examine episodic drinking and age of initiation of alcohol and drugs in relation to sexual behaviors	At the bivariate level, episodic but not non-episodic drinkers were more likely to have four or more lifetime sexual partners and more than one recent sexual partner compared to non-drinkers, among males and females. Condom use was higher for non-episodic drinkers, and lower for episodic drinkers, than non-drinkers, among males only. Controlling for demographics, episodic but not non-episodic drinkers were significantly more likely to have two or more lifetime sexual partners than non-drinkers. Drinking status was not correlated with condom use. Age at first drink was not correlated with condom use or multiple sexual partners.
Scroggins & Shacham (2021) [35]	Alcohol: Alcohol consumption pattern, based on frequency and amount of drinking in past 30 days (non-drinker, binge drank ≥ 1 days, drank with no binge drinking) HIV: Condom use at last intercourse (yes, no)	Definition: Described variables as “alcohol use and sexual risk behaviors”; analyzed alcohol use pattern as ordinal Analysis: Chi-square tests for bivariate associations between alcohol use pattern and condom use; univariate and multivariate logistic regressions to estimate odds of condom non-use based on alcohol-use pattern	Alcohol use was significantly associated with condom use in each analysis. In the adjusted model, those who drank with no binge drinking and those who reported no recent alcohol use were more likely to report condom use than those with at least one binge-drinking episode.
Thamotharan et al. (2015) [36]	Alcohol: Lifetime alcohol use (0, 1 or 2, 3–9, 10–19, 20–39, 40 or more days); alcohol use in past 30 days (0, 1 or 2, 3–5, 6–9, 10–19, 20–29, all 30 days); age of initiation for alcohol use (never, ≤11, 12, 13 … ≥17) HIV: Lifetime and past 3-month sexual partners (never, 1, 2… ≥6); condom use at last sexual intercourse (yes, no); ever being tested for HIV (yes, no).	Definition: Described variables as “substance use and sexual risk behaviors”; analyzed all multilevel variables as ordinal Analysis: Logistic and linear regression analyses to determine the relationship between alcohol use behaviors and sexual risk behaviors, including age and race/ethnicity as covariates	Greater lifetime and recent alcohol consumption both significantly correlated with more lifetime and recent sexual partners, not using a condom during recent sexual intercourse, and having ever been tested for HIV. Age of first alcohol use was not associated with number of lifetime or recent sexual partners or condom use.
Zhao et al. (2017) [37]	Alcohol: Alcohol use in past 30 days (≥1 vs. 0 days) HIV: Condom use at last intercourse (yes, no)	Definition: Referred to “alcohol use and sexual risk behaviors”; examined alcohol use as binary Analysis: Binary logistic regression models stratified by sex to examine condom use in relation to alcohol use and other variables; independent t and chi-square tests) to compare male and female respondents.	Recent alcohol drinking was not significantly associated with condomless sex for either males or females.

Note. HIV = human immunodeficiency virus. STD = sexually transmitted disease.

## Data Availability

No new data were created or analyzed in this study.

## Supplementary Materials

The following supporting information can be downloaded at: https://www.mdpi.com/article/10.3390/healthcare13182370/s1, Table S1: Quality Assessment of Included Studies.
