# Supplementary material for: Understanding the Relationship Between Alcohol Consumption and HIV Risk Behaviors in U.S. Adolescents: A Systematic Review of Youth Risk Behavior Survey Findings (2005–2021)
Source: Healthcare (Basel). 2025 Sep 21;13(18):2370. doi: 10.3390/healthcare13182370 (PMC12470159; doi:10.3390/healthcare13182370)
Supplement: Supplementary file 1 [file healthcare-13-02370-s001.zip › healthcare-3799762-supplementary.pdf]

Supplementary Table S1. Quality Assessment of Included Studies.

Author (year)	Q1	Q2	Q3	Q4	Q5	Q6	Q7	Q8	Q9	Q10	Q11	Q12	Q13	Q14
Ahmadi-Montecalvo et al. (2019)	Yes	Yes	Yes	Yes	Yes	No	No	No	Yes	NA	Yes	NA	NA	Yes
Aslam et al. (2023)	Yes	Yes	Yes	Yes	Yes	No	No	No	No	NA	No	NA	NA	Yes
Cavazos-Rehg et al. (2011)	Yes	Yes	Yes	Yes	Yes	No	No	Yes	Yes	NA	Yes	NA	NA	Yes
Clayton et al. (2019)	Yes	Yes	Yes	Yes	Yes	No	No	No	Yes	NA	Yes	NA	NA	Yes
Connell et al. (2009)	Yes	Yes	Yes	Yes	Yes	No	No	Yes	Yes	NA	Yes	NA	NA	Yes
Desai et al. (2021)	Yes	Yes	Yes	Yes	Yes	No	No	No	Yes	NA	Yes	NA	NA	Yes
Finch & Pierson (2011)	Yes	Yes	Yes	Yes	Yes	No	No	No	Yes	NA	Yes	NA	NA	Yes
Gao et al. (2017)	Yes	Yes	Yes	Yes	Yes	No	No	Yes	Yes	NA	Yes	NA	NA	Yes
Hingson & Zha (2018)	Yes	Yes	Yes	Yes	Yes	No	No	Yes	Yes	NA	Yes	NA	NA	Yes
Liddon et al. (2016)	Yes	Yes	Yes	Yes	Yes	No	No	No	Yes	NA	Yes	NA	NA	Yes
Mahat & Kelly (2023)	Yes	Yes	Yes	Yes	Yes	No	No	No	Yes	NA	Yes	NA	NA	Yes
McGuire et al. (2012)	Yes	Yes	Yes	Yes	Yes	No	No	No	No	NA	No	NA	NA	Yes
Outlaw et al. (2022)	Yes	Yes	Yes	Yes	Yes	No	No	No	Yes	NA	Yes	NA	NA	Yes
Ramisetty-Mikler & Ebama (2011)	Yes	Yes	Yes	Yes	Yes	No	No	Yes	Yes	NA	No	NA	NA	Yes
Scroggins & Shacham (2021)	Yes	Yes	Yes	Yes	Yes	No	No	Yes	Yes	NA	Yes	NA	NA	Yes
Thamotharan et al. (2014)	Yes	Yes	Yes	Yes	Yes	No	No	Yes	Yes	NA	Yes	NA	NA	Yes
Zhao et al. (2017)	Yes	Yes	Yes	Yes	Yes	No	No	No	Yes	NA	Yes	NA	NA	Yes

Note. All studies were assessed using the Quality Assessment Tool for Observational Cohort and Cross-Sectional Studies from the National Heart, Lung, and Blood Institute. The full set of questions read as follows:

Q1. Was the research question or objective in this paper clearly stated? .

Q2. Was the study population clearly specified and defined?

Q3. Was the participation rate of eligible persons at least 50%?

Q4. Were all the subjects selected or recruited from the same or similar populations (including the same time period)? Were inclusion and exclusion criteria for being in the study prespecified and applied uniformly to all participants?

Q5. Was a sample size justification, power description, or variance and effect estimates provided?

Q6. For the analyses in this paper, were the exposure(s) of interest measured prior to the outcome(s) being measured?
Q7. Was the timeframe sufficient so that one could reasonably expect to see an association between exposure and outcome if it existed?
Q8. For exposures that can vary in amount or level, did the study examine different levels of the exposure as related to the outcome (e.g., categories of exposure, or exposure measured as continuous variable)?
Q9. Were the exposure measures (independent variables) clearly defined, valid, reliable, and implemented consistently across all study participants?
Q10. Was the exposure(s) assessed more than once over time?
Q11. Were the outcome measures (dependent variables) clearly defined, valid, reliable, and implemented consistently across all study participants?
Q12. Were the outcome assessors blinded to the exposure status of participants?
Q13. Was loss to follow-up after baseline 20% or less?
Q14. Were key potential confounding variables measured and adjusted statistically for their impact on the relationship between exposure(s) and outcome(s)?
